# Crystal structure of [2-({2-[(2-azanidyl­benzyl­idene)amino]­benzyl­idene}amino)-4-chloro­phenol­ato]nickel(II)

**DOI:** 10.1107/S2056989017004613

**Published:** 2017-03-31

**Authors:** Fumiya Kobayashi, Atsushi Koga, Ryo Ohtani, Shinya Hayami, Masaaki Nakamura

**Affiliations:** a Department of Chemistry, Graduate School of Science and Technology, Kumamoto University, 2-39-1 Kurokami, Kumamoto 860-8555, Japan

**Keywords:** crystal structure, nickel(II) complex, 2-amino­benzaldehyde, asymmetric structure, supra­molecular structure

## Abstract

The title complex, [Ni(C_20_H_14_ClN_3_O)], with an asymmetrically chloride-appended Schiff base ligand has been synthesized and structurally characterized at 100 K. In the compound, the central nickel(II) ion has a square-planar coordination geometry with N_3_O donors of the π-conjugated tetra­dentate Schiff base ligand. In the crystal, the complexes are connected into an inversion dimer *via* an Ni⋯Ni inter­action [3.1753 (5) Å] and a pair of π–π inter­actions [centroid–centroid distance = 3.8416 (16) Å]. The dimers are linked *via* a C—H⋯Cl hydrogen bond, forming a chain along the *c*-axis direction. The dimer chains inter­act with each other through π–π inter­actions [centroid–centroid distance = 3.8736 (16) Å], forming a layer expanding parallel to the *ac* plane.

## Abstract 2

The metal atom of the title compound is four coordinated. The asymmetrically appended Cl atom and a widely spread π-conjugated system of the complex mol­ecule construct the supra­molecular structures of a hydrogen-bonded chain and a π–π inter­acted column.

## Chemical context   

Metal complexes with a tetra­dentate Schiff base ligand as represented by H_2_(salen) [*N*,*N*′-ethyl­enebis(salicyl­idene­imine)] and its derivatives have played extremely important roles in the field of coordination chemistry. Up to now, a large number of salen derivatives have been prepared and used for complexation in the expectation of a wide range of features such as catalytic ability, magnetic, dielectric and luminescence properties and so on (Bermejo *et al.*, 1996 ▸). In these cases, symmetric tetra­dentate ligands mainly produce N_2_O_2_ or N_4_ type coordination environments. In this research, we have designed asymmetric structures, both in the coordination environment and in the mol­ecular configuration, for the construction of the supra­molecular structure through inter­molecular hydrogen bonds, and synthesized the title nickel(II) complex using an asymmetrically chloride-appended tetra­dentate Schiff base ligand.
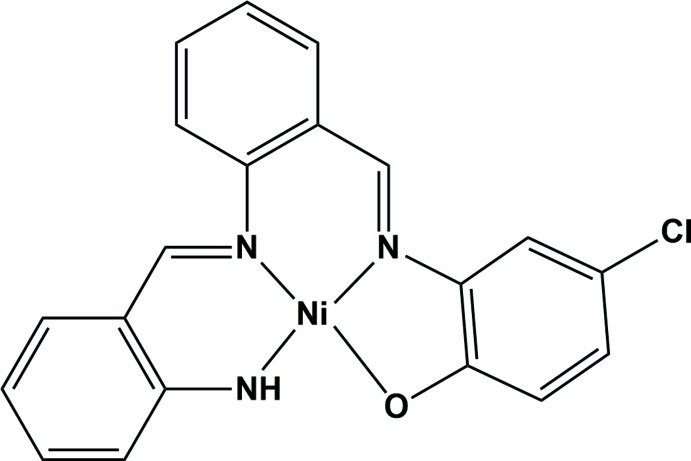


The structure of the title compound, which features a widely spread π-conjugated ring system, is also useful for supra­molecular assemblies through π–π inter­actions. The mononuclear copper(II) complex with a similar N_3_O type asymmetrical ligand was reported by Ghorai & Mukherjee (2014 ▸).

## Structural commentary   

**Figure 1:**
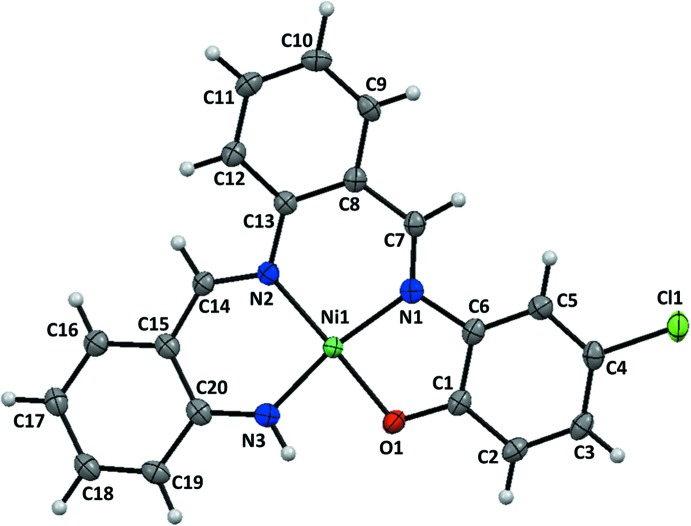
The mol­ecular structure of the title compound, showing displacement ellipsoids at the 50% probability level.

The nickel(II) atom is in a square-planar coordination with an asymmetrical coordination environment formed by the N_3_O donor set including one phenolate O atom, two imine N atoms and one amino N atom of the tetra­dentate Schiff base ligand (Fig. 1 ▸). The Ni—O1, Ni—N1, Ni—N2, and Ni—N3 bond lengths are 1.8617 (18), 1.878 (2), 1.896 (2) and 1.831 (2) Å, respectively. The complex mol­ecule is approximately planar; the coordination plane (N1–N3/O1/Ni1) makes dihedral angles of 4.15 (12), 10.22 (12) and 13.42 (12)°, respectively, with the C1–C6, C8–C13 and C15–C20 benzene rings.

## Supra­molecular features   

**Figure 2:**
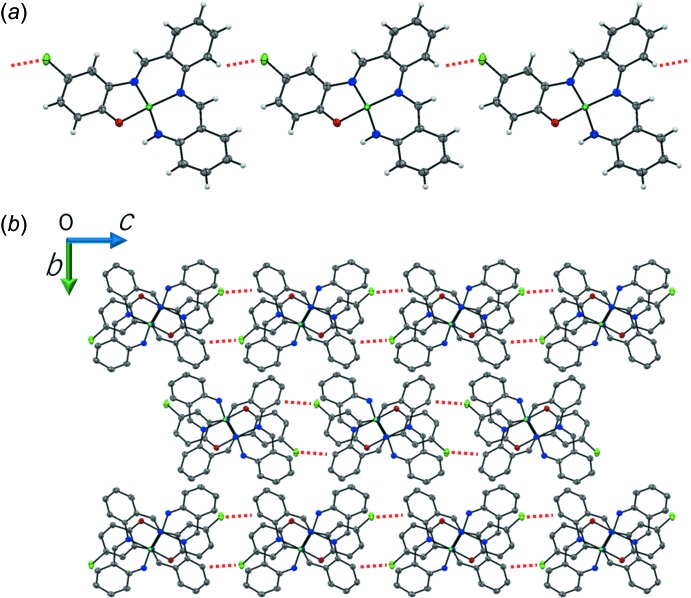
Packing diagrams of the title compound, showing (*a*) a chain structure running along the *c* axis formed by C—H⋯Cl hydrogen bonds (red dashed lines) and (*b*) the chains viewed along the *a* axis.

**Figure 3:**
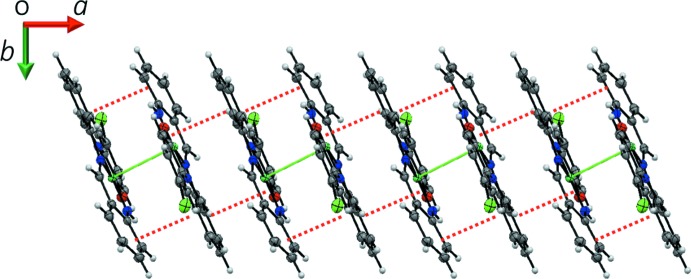
A packing diagram of the title compound, showing the column structure along the *a* axis formed by Ni⋯Ni inter­actions (green solid lines) and π–π inter­actions (red dashed lines).

**Figure 4:**
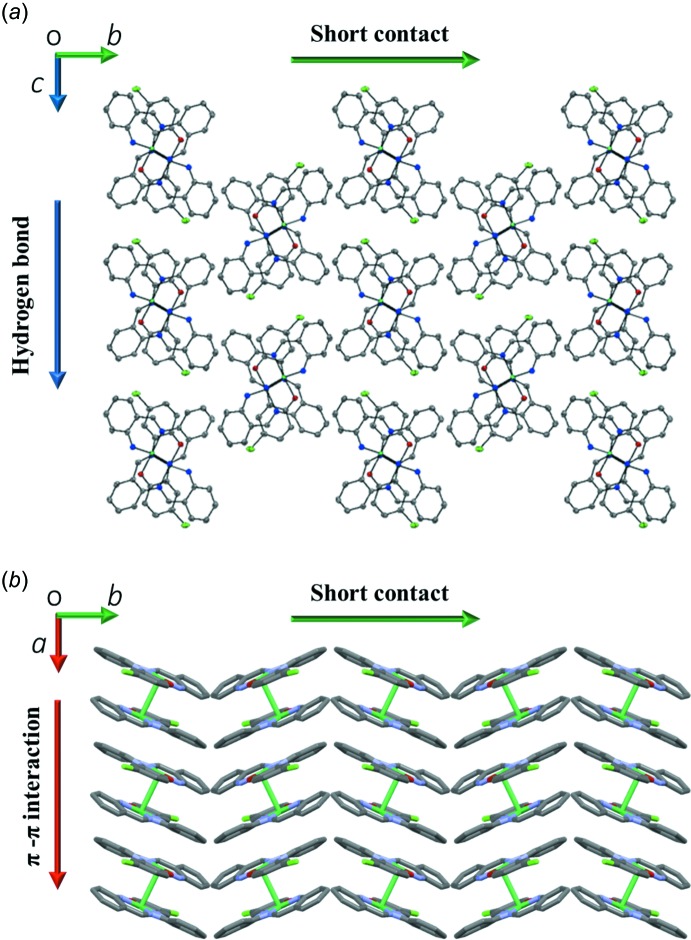
Packing diagrams of the title compound assembled by (*a*) C—H⋯Cl hydrogen bonds and C—H⋯C short contacts, and (*b*) π–π inter­actions and short contacts.

In the crystal, pairs of complex mol­ecules related by an inversion centre are dimerized by an Ni⋯Ni inter­action [3.1753 (5) Å] and a pair of π–π inter­actions between the C1–C6 and C15–C20 benzene rings [centroid–centroid distance = 3.8415 (16) Å]. Such dimerization caused by an Ni⋯Ni inter­action has also been observed in symmetric Ni(salen) compounds (Aullón *et al.*, 1996 ▸; Siegler & Lutz, 2009 ▸). The dimeric mol­ecules of the title compound are linked by C—H⋯Cl hydrogen bonds (Table 1 ▸), producing a chain of dimers along the *c* axis (Fig. 2 ▸). The dimers further inter­act with each other through π–π inter­actions between the C1–C6 and C8–C13 benzene rings [centroid–centroid distance = 3.8738 (17) Å], forming a column along the *a* axis (Fig. 3 ▸). Together, these C—H⋯Cl and π–π inter­actions result in a layer parallel to the *ac* plane. The layers are further linked by a short C—H⋯C contact (Table 1 ▸), giving a three-dimensional network (Fig. 4 ▸).

## Synthesis and crystallization   

The tetra­dentate Schiff base ligand was prepared by the reaction of 2-amino­benzaldehyde (Smith & Opie, 1948 ▸) (0.228 g, 2.0 mmol) and 2-amino-4-chloro­phenol (0.144 g, 1.0 mmol) in methanol (50 ml) under stirring for 1 h. The resulting solution including the ligand was used for complexation with the Ni^II^ ion. A methanol solution (50 ml) of Ni(CH_3_COO)_2_·4H_2_O (0.249 g, 1.0 mmol) was added to the solution and stirred for 1 h. The resulting solution was allowed to stand for a few days, during which time dark-purple block-shaped crystals precipitated. They were collected by suction filtration and dried in air to give single crystals of the title compound suitable for X-ray diffraction.

## Refinement   

Crystal data, data collection and structure refinement details are summarized in Table 2 ▸. The position of the N-bound H atom was refined with N—H = 0.86 (1) Å and *U*_iso_(H) = 1.5*U*_eq_(N). Other H atoms were treated as riding with C—H = 0.95 Å and *U*_iso_(H) = 1.2*U*_eq_(C).

## Supplementary Material

Crystal structure: contains datablock(s) global, I. DOI: 10.1107/S2056989017004613/is5472sup1.cif

Structure factors: contains datablock(s) I. DOI: 10.1107/S2056989017004613/is5472Isup2.hkl

CCDC reference: 1539785

Additional supporting information:  crystallographic information; 3D view; checkCIF report

## Figures and Tables

**Table 1:** Hydrogen-bond geometry (Å, °)

*D*—H⋯*A*	*D*—H	H⋯*A*	*D*⋯*A*	*D*—H⋯*A*
C12—H12⋯Cl1^i^	0.95	2.76	3.540 (3)	140
C10—H10⋯C20^ii^	0.95	2.80	3.626 (4)	146

Symmetry codes: (i) 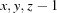; (ii) 
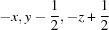
.

**Table 2:** Experimental details

Crystal data	
Chemical formula	[Ni(C_20_H_14_ClN_3_O)]
*M*_r_	406.50
Crystal system, space group	Monoclinic, *P*2_1_/*c*
Temperature (K)	100
*a*, *b*, *c* (Å)	7.5510 (4), 17.8689 (9), 12.6834 (6)
β (°)	109.9504 (14)
*V* (Å^3^)	1608.64 (14)
*Z*	4
Radiation type	Mo *K*α
μ (mm^−1^)	1.39
Crystal size (mm)	0.46 × 0.27 × 0.25

Data collection	
Diffractometer	Rigaku R-AXIS RAPID
Absorption correction	Multi-scan (*ABSCOR*; Higashi, 1995 ▸)
*T*_min_, *T*_max_	0.476, 0.712
No. of measured, independent and observed [*F* ^2^ > 2.0σ(*F* ^2^)] reflections	15243, 3638, 3177
*R*_int_	0.039
(sin θ/λ)_max_ (Å^−1^)	0.647

Refinement	
*R*[*F* ^2^ > 2σ(*F* ^2^)], *wR*(*F* ^2^), *S*	0.042, 0.103, 1.04
No. of reflections	3638
No. of parameters	238
No. of restraints	1
H-atom treatment	H atoms treated by a mixture of independent and constrained refinement
Δρ_max_, Δρ_min_ (e Å^−3^)	1.35, −0.37

Computer programs: *RAPID-AUTO* (Rigaku, 1995 ▸), *SHELXS2013* (Sheldrick, 2008 ▸), *SHELXL2016* (Sheldrick, 2015 ▸) and *CrystalStructure* (Rigaku, 2014 ▸).

## supplementary crystallographic information

### Crystal data

**Table:**

[Ni(C_20_H_14_ClN_3_O)]	*F*(000) = 832.00
*M**_r_* = 406.50	*D*_x_ = 1.678 Mg m^−^^3^
Monoclinic, *P*2_1_/*c*	Mo *K*α radiation, λ = 0.71075 Å
*a* = 7.5510 (4) Å	Cell parameters from 13442 reflections
*b* = 17.8689 (9) Å	θ = 3.0–27.4°
*c* = 12.6834 (6) Å	µ = 1.39 mm^−^^1^
β = 109.9504 (14)°	*T* = 100 K
*V* = 1608.64 (14) Å^3^	Block, purple
*Z* = 4	0.46 × 0.27 × 0.25 mm

### Data collection

**Table:**

Rigaku R-AXIS RAPID diffractometer	3177 reflections with *F*^2^ > 2.0σ(*F*^2^)
Detector resolution: 10.000 pixels mm^-1^	*R*_int_ = 0.039
ω scans	θ_max_ = 27.4°, θ_min_ = 3.0°
Absorption correction: multi-scan (ABSCOR; Higashi, 1995)	*h* = −9→8
*T*_min_ = 0.476, *T*_max_ = 0.712	*k* = −23→23
15243 measured reflections	*l* = −16→16
3638 independent reflections	

### Refinement

**Table:**

Refinement on *F*^2^	Secondary atom site location: difference Fourier map
*R*[*F*^2^ > 2σ(*F*^2^)] = 0.042	Hydrogen site location: inferred from neighbouring sites
*wR*(*F*^2^) = 0.103	H atoms treated by a mixture of independent and constrained refinement
*S* = 1.04	*w* = 1/[σ^2^(*F*_o_^2^) + (0.0527*P*)^2^ + 1.948*P*] where *P* = (*F*_o_^2^ + 2*F*_c_^2^)/3
3638 reflections	(Δ/σ)_max_ = 0.001
238 parameters	Δρ_max_ = 1.35 e Å^−^^3^
1 restraint	Δρ_min_ = −0.37 e Å^−^^3^
Primary atom site location: structure-invariant direct methods	

### Special details

**Table:**

Geometry. All esds (except the esd in the dihedral angle between two l.s. planes) are estimated using the full covariance matrix. The cell esds are taken into account individually in the estimation of esds in distances, angles and torsion angles; correlations between esds in cell parameters are only used when they are defined by crystal symmetry. An approximate (isotropic) treatment of cell esds is used for estimating esds involving l.s. planes.
Refinement. Refinement was performed using all reflections. The weighted R-factor (wR) and goodness of fit (S) are based on F^2^. R-factor (gt) are based on F. The threshold expression of F^2^ > 2.0 sigma(F^2^) is used only for calculating R-factor (gt).

### Fractional atomic coordinates and isotropic or equivalent isotropic displacement parameters (Å^2^)

**Table:**

	*x*	*y*	*z*	*U*_iso_*/*U*_eq_	
Ni1	0.29838 (4)	0.53846 (2)	0.46051 (3)	0.01597 (11)	
Cl1	0.22371 (10)	0.39200 (4)	0.93185 (6)	0.03146 (17)	
O1	0.3645 (3)	0.58686 (10)	0.59854 (15)	0.0230 (4)	
N1	0.2079 (3)	0.46083 (11)	0.52811 (18)	0.0195 (4)	
N2	0.2225 (3)	0.49436 (12)	0.31594 (18)	0.0193 (4)	
N3	0.3976 (3)	0.62220 (12)	0.41867 (19)	0.0221 (4)	
C1	0.3301 (4)	0.54570 (14)	0.6762 (2)	0.0205 (5)	
C2	0.3738 (4)	0.56917 (15)	0.7885 (2)	0.0229 (5)	
C3	0.3380 (4)	0.52243 (15)	0.8656 (2)	0.0237 (5)	
C4	0.2594 (4)	0.45198 (15)	0.8321 (2)	0.0236 (5)	
C5	0.2109 (4)	0.42792 (15)	0.7223 (2)	0.0224 (5)	
C6	0.2447 (4)	0.47561 (15)	0.6445 (2)	0.0207 (5)	
C7	0.1169 (4)	0.40146 (14)	0.4813 (2)	0.0210 (5)	
C8	0.0936 (3)	0.37840 (14)	0.3689 (2)	0.0197 (5)	
C9	0.0092 (4)	0.30739 (15)	0.3366 (2)	0.0235 (5)	
C10	−0.0089 (4)	0.27609 (15)	0.2338 (2)	0.0259 (6)	
C11	0.0649 (4)	0.31435 (15)	0.1634 (2)	0.0251 (5)	
C12	0.1469 (4)	0.38395 (15)	0.1918 (2)	0.0230 (5)	
C13	0.1542 (3)	0.41975 (13)	0.2923 (2)	0.0187 (5)	
C14	0.2147 (4)	0.53457 (14)	0.2259 (2)	0.0199 (5)	
C15	0.2954 (4)	0.60452 (14)	0.2207 (2)	0.0214 (5)	
C16	0.2781 (4)	0.63380 (15)	0.1133 (2)	0.0247 (5)	
C17	0.3660 (4)	0.69883 (15)	0.1023 (2)	0.0262 (5)	
C18	0.4758 (4)	0.73809 (15)	0.2007 (2)	0.0267 (6)	
C19	0.4922 (4)	0.71300 (14)	0.3054 (2)	0.0253 (6)	
C20	0.3979 (3)	0.64586 (14)	0.3194 (2)	0.0210 (5)	
H1	0.452 (4)	0.6459 (17)	0.478 (2)	0.0315*	
H2	0.42785	0.61711	0.81103	0.0275*	
H3	0.36681	0.5382	0.94116	0.0285*	
H5	0.15584	0.38005	0.70055	0.0268*	
H7	0.06228	0.37131	0.52382	0.0253*	
H9	−0.03614	0.28057	0.38682	0.0282*	
H10	−0.07053	0.22937	0.21193	0.0310*	
H11	0.05913	0.2923	0.09418	0.0301*	
H12	0.19936	0.40819	0.14264	0.0276*	
H14	0.14485	0.51283	0.15561	0.0239*	
H16	0.2041	0.60767	0.04782	0.0296*	
H17	0.35399	0.71755	0.03007	0.0314*	
H18	0.53905	0.7827	0.19338	0.0321*	
H19	0.56662	0.74021	0.36959	0.0304*	

### Atomic displacement parameters (Å^2^)

**Table:**

	*U*^11^	*U*^22^	*U*^33^	*U*^12^	*U*^13^	*U*^23^
Ni1	0.01684 (17)	0.01610 (17)	0.01561 (17)	0.00035 (11)	0.00636 (13)	0.00011 (12)
Cl1	0.0320 (4)	0.0439 (4)	0.0214 (3)	−0.0047 (3)	0.0130 (3)	0.0036 (3)
O1	0.0285 (10)	0.0222 (9)	0.0181 (9)	0.0016 (8)	0.0076 (8)	−0.0014 (7)
N1	0.0167 (10)	0.0209 (11)	0.0216 (11)	0.0035 (8)	0.0072 (9)	0.0015 (8)
N2	0.0177 (10)	0.0202 (10)	0.0215 (10)	0.0012 (8)	0.0088 (9)	0.0001 (8)
N3	0.0210 (11)	0.0203 (11)	0.0238 (11)	0.0001 (9)	0.0062 (9)	−0.0002 (9)
C1	0.0183 (12)	0.0243 (13)	0.0183 (12)	0.0059 (10)	0.0054 (10)	0.0001 (10)
C2	0.0230 (12)	0.0228 (12)	0.0200 (12)	0.0049 (10)	0.0036 (10)	−0.0025 (10)
C3	0.0204 (12)	0.0315 (14)	0.0190 (12)	0.0063 (11)	0.0064 (10)	−0.0022 (11)
C4	0.0198 (12)	0.0304 (14)	0.0222 (13)	0.0036 (10)	0.0092 (10)	0.0040 (11)
C5	0.0180 (12)	0.0254 (13)	0.0241 (13)	0.0006 (10)	0.0078 (10)	−0.0024 (11)
C6	0.0171 (11)	0.0261 (13)	0.0196 (12)	0.0047 (10)	0.0070 (10)	−0.0001 (10)
C7	0.0188 (12)	0.0246 (13)	0.0211 (12)	0.0017 (10)	0.0085 (10)	0.0024 (10)
C8	0.0160 (11)	0.0228 (12)	0.0197 (12)	0.0032 (9)	0.0055 (10)	0.0023 (10)
C9	0.0195 (12)	0.0238 (13)	0.0276 (13)	−0.0001 (10)	0.0085 (11)	0.0055 (11)
C10	0.0218 (13)	0.0217 (12)	0.0310 (14)	−0.0024 (10)	0.0049 (11)	−0.0047 (11)
C11	0.0241 (13)	0.0267 (13)	0.0221 (13)	0.0019 (11)	0.0048 (11)	−0.0039 (11)
C12	0.0252 (13)	0.0232 (13)	0.0206 (12)	0.0038 (10)	0.0077 (11)	0.0003 (10)
C13	0.0167 (11)	0.0183 (12)	0.0209 (12)	0.0025 (9)	0.0062 (10)	0.0012 (10)
C14	0.0189 (12)	0.0216 (12)	0.0203 (12)	0.0019 (10)	0.0080 (10)	−0.0009 (10)
C15	0.0207 (12)	0.0182 (12)	0.0279 (13)	0.0029 (10)	0.0115 (11)	0.0015 (10)
C16	0.0242 (13)	0.0246 (13)	0.0274 (14)	0.0022 (11)	0.0116 (11)	−0.0010 (11)
C17	0.0302 (14)	0.0236 (13)	0.0291 (14)	0.0032 (11)	0.0158 (12)	0.0052 (11)
C18	0.0273 (13)	0.0204 (12)	0.0364 (15)	0.0002 (11)	0.0160 (12)	0.0038 (11)
C19	0.0216 (12)	0.0211 (13)	0.0330 (15)	−0.0002 (10)	0.0087 (11)	0.0007 (11)
C20	0.0165 (11)	0.0193 (12)	0.0285 (13)	0.0055 (9)	0.0094 (10)	0.0044 (10)

### Geometric parameters (Å, º)

**Table:**

Ni1—N3	1.831 (2)	C8—C13	1.416 (3)
Ni1—O1	1.8617 (18)	C8—C9	1.416 (4)
Ni1—N1	1.878 (2)	C9—C10	1.382 (4)
Ni1—N2	1.896 (2)	C9—H9	0.9500
Cl1—C4	1.748 (3)	C10—C11	1.383 (4)
O1—C1	1.324 (3)	C10—H10	0.9500
N1—C7	1.293 (3)	C11—C12	1.381 (4)
N1—C6	1.430 (3)	C11—H11	0.9500
N2—C14	1.333 (3)	C12—C13	1.410 (3)
N2—C13	1.424 (3)	C12—H12	0.9500
N3—C20	1.329 (3)	C14—C15	1.402 (3)
N3—H1	0.836 (18)	C14—H14	0.9500
C1—C6	1.403 (4)	C15—C16	1.424 (4)
C1—C2	1.412 (3)	C15—C20	1.432 (4)
C2—C3	1.381 (4)	C16—C17	1.369 (4)
C2—H2	0.9500	C16—H16	0.9500
C3—C4	1.395 (4)	C17—C18	1.425 (4)
C3—H3	0.9500	C17—H17	0.9500
C4—C5	1.382 (4)	C18—C19	1.367 (4)
C5—C6	1.392 (4)	C18—H18	0.9500
C5—H5	0.9500	C19—C20	1.436 (4)
C7—C8	1.436 (3)	C19—H19	0.9500
C7—H7	0.9500		
			
N3—Ni1—O1	83.57 (9)	C13—C8—C7	125.0 (2)
N3—Ni1—N1	169.92 (10)	C9—C8—C7	115.8 (2)
O1—Ni1—N1	86.35 (9)	C10—C9—C8	121.7 (2)
N3—Ni1—N2	94.46 (10)	C10—C9—H9	119.1
O1—Ni1—N2	176.53 (9)	C8—C9—H9	119.1
N1—Ni1—N2	95.61 (9)	C9—C10—C11	118.5 (2)
C1—O1—Ni1	112.57 (16)	C9—C10—H10	120.8
C7—N1—C6	120.7 (2)	C11—C10—H10	120.8
C7—N1—Ni1	128.02 (18)	C12—C11—C10	121.4 (3)
C6—N1—Ni1	111.23 (16)	C12—C11—H11	119.3
C14—N2—C13	114.6 (2)	C10—C11—H11	119.3
C14—N2—Ni1	121.00 (18)	C11—C12—C13	121.3 (2)
C13—N2—Ni1	124.14 (16)	C11—C12—H12	119.3
C20—N3—Ni1	131.89 (19)	C13—C12—H12	119.3
C20—N3—H1	122 (2)	C12—C13—C8	117.5 (2)
Ni1—N3—H1	106 (2)	C12—C13—N2	121.0 (2)
O1—C1—C6	118.0 (2)	C8—C13—N2	121.5 (2)
O1—C1—C2	123.2 (2)	N2—C14—C15	128.9 (2)
C6—C1—C2	118.7 (2)	N2—C14—H14	115.6
C3—C2—C1	120.0 (3)	C15—C14—H14	115.6
C3—C2—H2	120.0	C14—C15—C16	118.3 (2)
C1—C2—H2	120.0	C14—C15—C20	122.2 (2)
C2—C3—C4	119.7 (2)	C16—C15—C20	119.5 (2)
C2—C3—H3	120.1	C17—C16—C15	121.3 (3)
C4—C3—H3	120.1	C17—C16—H16	119.3
C5—C4—C3	121.8 (2)	C15—C16—H16	119.3
C5—C4—Cl1	119.0 (2)	C16—C17—C18	119.1 (3)
C3—C4—Cl1	119.2 (2)	C16—C17—H17	120.5
C4—C5—C6	118.3 (2)	C18—C17—H17	120.5
C4—C5—H5	120.8	C19—C18—C17	121.5 (2)
C6—C5—H5	120.8	C19—C18—H18	119.3
C5—C6—C1	121.4 (2)	C17—C18—H18	119.3
C5—C6—N1	126.9 (2)	C18—C19—C20	120.6 (3)
C1—C6—N1	111.7 (2)	C18—C19—H19	119.7
N1—C7—C8	123.8 (2)	C20—C19—H19	119.7
N1—C7—H7	118.1	N3—C20—C15	119.2 (2)
C8—C7—H7	118.1	N3—C20—C19	122.9 (2)
C13—C8—C9	119.2 (2)	C15—C20—C19	117.8 (2)
			
N3—Ni1—O1—C1	−176.59 (18)	Ni1—N1—C7—C8	10.2 (4)
N1—Ni1—O1—C1	3.42 (17)	N1—C7—C8—C13	−4.8 (4)
N3—Ni1—N1—C7	173.9 (5)	N1—C7—C8—C9	172.8 (2)
O1—Ni1—N1—C7	174.0 (2)	C13—C8—C9—C10	2.6 (4)
N2—Ni1—N1—C7	−3.1 (2)	C7—C8—C9—C10	−175.2 (2)
N3—Ni1—N1—C6	−3.6 (6)	C8—C9—C10—C11	2.7 (4)
O1—Ni1—N1—C6	−3.50 (16)	C9—C10—C11—C12	−3.2 (4)
N2—Ni1—N1—C6	179.37 (16)	C10—C11—C12—C13	−1.7 (4)
N3—Ni1—N2—C14	−15.0 (2)	C11—C12—C13—C8	6.9 (4)
N1—Ni1—N2—C14	164.51 (19)	C11—C12—C13—N2	−173.9 (2)
N3—Ni1—N2—C13	170.91 (19)	C9—C8—C13—C12	−7.2 (3)
N1—Ni1—N2—C13	−9.6 (2)	C7—C8—C13—C12	170.3 (2)
O1—Ni1—N3—C20	−171.4 (2)	C9—C8—C13—N2	173.6 (2)
N1—Ni1—N3—C20	−171.3 (4)	C7—C8—C13—N2	−8.9 (4)
N2—Ni1—N3—C20	5.7 (2)	C14—N2—C13—C12	22.3 (3)
Ni1—O1—C1—C6	−2.6 (3)	Ni1—N2—C13—C12	−163.25 (19)
Ni1—O1—C1—C2	177.86 (19)	C14—N2—C13—C8	−158.5 (2)
O1—C1—C2—C3	−178.4 (2)	Ni1—N2—C13—C8	15.9 (3)
C6—C1—C2—C3	2.1 (4)	C13—N2—C14—C15	−169.2 (2)
C1—C2—C3—C4	0.3 (4)	Ni1—N2—C14—C15	16.1 (4)
C2—C3—C4—C5	−1.8 (4)	N2—C14—C15—C16	175.5 (2)
C2—C3—C4—Cl1	177.5 (2)	N2—C14—C15—C20	−2.7 (4)
C3—C4—C5—C6	0.9 (4)	C14—C15—C16—C17	−174.8 (2)
Cl1—C4—C5—C6	−178.37 (19)	C20—C15—C16—C17	3.5 (4)
C4—C5—C6—C1	1.5 (4)	C15—C16—C17—C18	−0.4 (4)
C4—C5—C6—N1	178.9 (2)	C16—C17—C18—C19	−1.3 (4)
O1—C1—C6—C5	177.4 (2)	C17—C18—C19—C20	−0.2 (4)
C2—C1—C6—C5	−3.0 (4)	Ni1—N3—C20—C15	4.8 (4)
O1—C1—C6—N1	−0.2 (3)	Ni1—N3—C20—C19	−177.35 (19)
C2—C1—C6—N1	179.3 (2)	C14—C15—C20—N3	−8.6 (4)
C7—N1—C6—C5	7.7 (4)	C16—C15—C20—N3	173.2 (2)
Ni1—N1—C6—C5	−174.6 (2)	C14—C15—C20—C19	173.4 (2)
C7—N1—C6—C1	−174.8 (2)	C16—C15—C20—C19	−4.8 (3)
Ni1—N1—C6—C1	2.9 (3)	C18—C19—C20—N3	−174.7 (2)
C6—N1—C7—C8	−172.5 (2)	C18—C19—C20—C15	3.2 (4)

### Hydrogen-bond geometry (Å, º)

**Table:**

*D*—H···*A*	*D*—H	H···*A*	*D*···*A*	*D*—H···*A*
C12—H12···Cl1^i^	0.95	2.76	3.540 (3)	140
C10—H10···C20^ii^	0.95	2.80	3.626 (4)	146

Symmetry codes: (i) *x*, *y*, *z*−1; (ii) −*x*, *y*−1/2, −*z*+1/2.
